# *tert*-Butyl *N*-{[5-(5-oxohexa­namido)­pyridin-2-yl]amino}­carbamate

**DOI:** 10.1107/S1600536813024598

**Published:** 2013-09-12

**Authors:** Luisa Ronga, Noel Pinaud, Charlotte Rimbault, Mathieu Marchivie, Jean Guillon

**Affiliations:** a Université Bordeaux Segalen, CNRS FRE 3396 -Pharmacochimie, F-33076 Bordeaux, France; b ISM–CNRS UMR 5255, Université de Bordeaux, F-33405 Talence cedex, France

## Abstract

In the crystal structure of the title compound, C_16_H_24_N_4_O_4_, mol­ecules are linked by N—H⋯O hydrogen bonds between the carbonyl groups of the carbamoyl and amido functional groups and the amino groups, and by N—H⋯N hydrogen bonds between the amino group and the pyridine ring, forming two-dimensional networks parallel to the *ab* plane.

## Related literature

For the synthesis, properties and biological activity of 2-hydrazino­pyridine derivatives, see: Ardisson *et al.* (2005 ▶); Jurisson & Lydon (1999 ▶); Abrams *et al.* (1994 ▶); Liu *et al.* (2011 ▶); Lu *et al.* (2011 ▶); Schwartz *et al.* (1990 ▶). For the crystal structures of related compounds, see: Banerjee *et al.* (2005 ▶); Rose *et al.* (1998 ▶); Zora *et al.* (2006 ▶). For synthesis, see: Cugola *et al.* (1995 ▶).
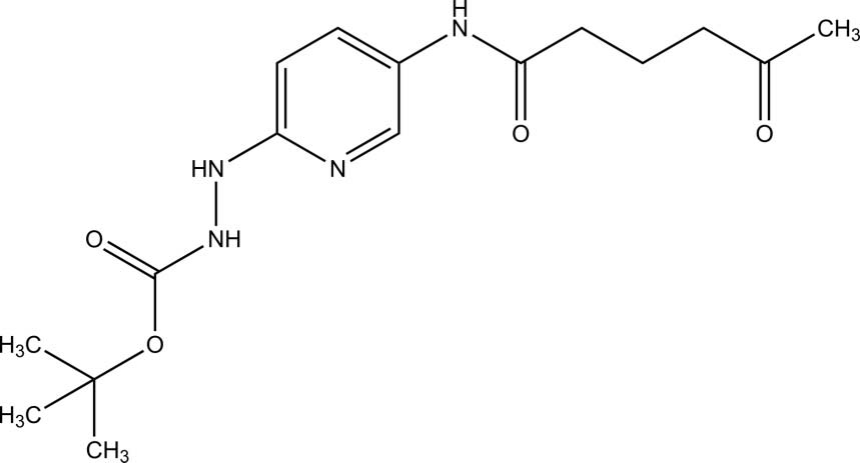


## Experimental

### 

#### Crystal data

C_16_H_24_N_4_O_4_
*M* *_r_* = 336.39
Triclinic, 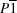

*a* = 6.2598 (4) Å
*b* = 9.2822 (6) Å
*c* = 16.0437 (12) Å
α = 84.387 (6)°
β = 88.957 (6)°
γ = 79.358 (6)°
*V* = 911.79 (11) Å^3^
*Z* = 2
Mo *K*α radiation
μ = 0.09 mm^−1^
*T* = 293 K
0.84 × 0.17 × 0.06 mm

#### Data collection

Bruker–Nonius KappaCCD diffractometer
Absorption correction: multi-scan (*SADABS*; Sheldrick, 1996 ▶) *T*_min_ = 0.928, *T*_max_ = 0.994
20299 measured reflections
3295 independent reflections
2187 reflections with *I* > 2σ(*I*)
*R*_int_ = 0.038

#### Refinement

*R*[*F* ^2^ > 2σ(*F* ^2^)] = 0.053
*wR*(*F* ^2^) = 0.147
*S* = 1.04
3295 reflections
221 parameters
H-atom parameters constrained
Δρ_max_ = 0.29 e Å^−3^
Δρ_min_ = −0.25 e Å^−3^

### 

Data collection: *COLLECT* (Nonius, 1998 ▶); cell refinement: *DIRAX/LSQ* (Duisenberg, 1992 ▶); data reduction: *EVALCCD* (Duisenberg *et al.*, 2003 ▶); program(s) used to solve structure: *SHELXS97* (Sheldrick, 2008 ▶); program(s) used to refine structure: *SHELXL97* (Sheldrick, 2008 ▶); molecular graphics: *OLEX2* (Dolomanov *et al.*, 2009 ▶); software used to prepare material for publication: *publCIF* (Westrip, 2010 ▶).

## Supplementary Material

Crystal structure: contains datablock(s) I. DOI: 10.1107/S1600536813024598/ff2115sup1.cif

Structure factors: contains datablock(s) I. DOI: 10.1107/S1600536813024598/ff2115Isup2.hkl

Supplementary material file. DOI: 10.1107/S1600536813024598/ff2115Isup3.cml

Additional supplementary materials:  crystallographic information; 3D view; checkCIF report

## Figures and Tables

**Table 1:** Hydrogen-bond geometry (Å, °)

*D*—H⋯*A*	*D*—H	H⋯*A*	*D*⋯*A*	*D*—H⋯*A*
N1—H1⋯O3^i^	0.86	2.06	2.888 (2)	161
N3—H3⋯N2^ii^	0.86	2.21	2.957 (3)	145
N4—H4⋯O2^iii^	0.86	2.06	2.827 (3)	149

Symmetry codes: (i) 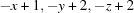; (ii) 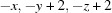; (iii) 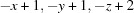.

**Fig. 1.**
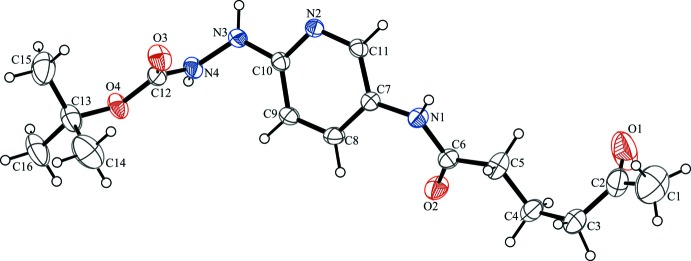
The molecular structure of the title compound. Displacement ellipsoids are drawn at the 30% probability level.

**Fig. 2.**
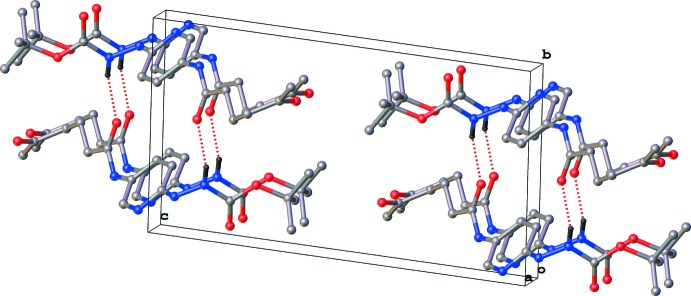
Crystal packing of the title compound viewed down the a axis. Only hydrogen atoms involved in hydrogen bonding (dashed lines) are shown.

## supplementary crystallographic information

### 1. Comment

Radioisotopes conjugated to proteins provide a means for imaging and treatment of disease. The bifunctional 2-hydrazinopyridine derivatives are useful linker molecules for attaching metal ions such as ^99^*^m^*Tc to macromolecules (Ardisson *et al.*, 2005; Jurisson & Lydon, 1999). Hence, this 2-hydrazinopyridinyl moiety has previously been used for labeling bioactive molecules (Abrams *et al.*, 1994; Banerjee *et al.* 2005; Rose *et al.*, 1998; Schwartz *et al.*, 1990). Thus, the use of Tc-labeled hydrazine derivatives continues to undergo further development (Liu *et al.*, 2011; Lu *et al.*, 2011). The wide spectrum of medicinal applications of this class of radiolabeled chelates prompted us to work in this domain and we report herein on the synthesis and crystal structure of the title compound, designed as a potential chelate for ^99^*^m^*Tc.

The title compound, C_16_H_24_N_4_O_4_, has the triclinic (*P*1) symmetry. It crystalizes with one molecule in the asymmetric unit. In the crystal, the molecules are linked together by N—H···O hydrogen bonding between the carbonyl groups of the carbamoyl and amido functional groups and the amino groups and by N—H···N hydrogen bonding between amino and pyridine moiety leading to a two-dimensional network within the *ab* plane. The network cohesion in the 3^rd^ direction is assured by Van der Waals interactions and H-bond like interactions between the carbonyl and the BOC group.

### 2. Experimental

To a stirred solution of 5-oxohexanoic acid (2.45 mmol) in 15 ml of tetrahydrofurane was added triethylamine (2.45 mmol). After 10 min of stirring at room temperature was added isobutyl chloroformate (2.45 mmol). The reaction mixture was stirred at room temperature for 5 h, then 2-(*t*-butoxycarbonyl hydrazine)-5-amino-pyridine (2.23 mmol) (Cugola *et al.*,19955) was added and the reaction stirred for 12 h. The mixture was evaporated to dryness, and the residue was triturated in water. The solid precipitate was filtered off and washed with water then with ethanol, and purified by column chromatography using CHCl_3_/methanol (9/1, *v*/*v*) as eluent to give the title compound as white crystals (*R*_f_ = 1/4). Yield is 48%. The single-crystal of the title product was obtained by slow crystallization from a mixture DMSO/methanol (9/1, *v*/*v*). *M*.p. = 219°C. IR (KBr), ν/cm^-1^: 3270, 3230, 3185, 3082, 2982, 1704, 1662, 1601, 1537, 1276, 1182. ^1^H NMR (300 MHz, DMSO-d_6_, 298 K): δ = 1.41 (s, 9H, 3 CH_3_), 1.75 (qt, 2H, J = 6.10, CH_2_), 2.09 (s, 3H, CH_3_), 2.26 (t, 2H, J = 6.10, CH_2_), 2.48 (t, 2H, J = 6.10, CH_2_), 6.49 (d, 1H, J = 7.50, H-3), 7.71 (dd, 1H, J = 7.50 and 1.55, H-4), 7.95 (s, 1H, NH), 8.20 (d, 1H, J = 1.55, H-6), 8.75 (s, 1H, NH), 9.70 (s, 1H, NH). Anal. Calcd. for C_16_H_24_N_4_O_4_: C, 57.13; H, 7.19; N, 16.66 Found: C, 57.26; H, 7.25; N, 16.52.

### 3. Refinement

Crystallographic data were collected at 293 K on a Brucker nonius k-CCD diffractometer with monochromatic Mo—Kα radiation (λ = 0.71073 Å). At 293 K, the full sphere data collection was performed using φ scans and ω scans. The unit cell determination and data reduction were performed using *DIRAX/LSQ* (Duisenberg, 1992) and Collect (Nonius, 1998) programs on the full set of data. The crystal structure was solved by direct methods and successive Fourier difference syntheses with *SHELXS97* program (Sheldrick, 2008). The refinements of the crystal structure were performed on F2 by weighted anisotropic full-matrix least squares methods using the *SHELXL97* program (Sheldrick, 2008). Both pieces of software were used within OLEX2 package (Dolomanov *et al.*, 2009). All the non-H atoms were refined with anisotropic temperature parameters. The positions of the H atoms were deduced from coordinates of the non-H atoms and confirmed by Fourier synthesis and treated according to the riding model during refinement with isotropic displacement parameters, corresponding to the atom they are linked to. H atoms were included for structure factor calculations but not refined.

### Crystal data

**Table:**

C_16_H_24_N_4_O_4_	*Z* = 2
*M**_r_* = 336.39	*F*(000) = 360
Triclinic, *P*1	*D*_x_ = 1.225 Mg m^−^^3^
*a* = 6.2598 (4) Å	Mo *K*α radiation, λ = 0.71073 Å
*b* = 9.2822 (6) Å	Cell parameters from 8003 reflections
*c* = 16.0437 (12) Å	θ = 3.2–25.3°
α = 84.387 (6)°	µ = 0.09 mm^−^^1^
β = 88.957 (6)°	*T* = 293 K
γ = 79.358 (6)°	Plate, colourless
*V* = 911.79 (11) Å^3^	0.84 × 0.17 × 0.06 mm

### Data collection

**Table:**

Bruker–Nonius KappaCCD diffractometer	2187 reflections with *I* > 2σ(*I*)
intensities from φ scan and ω scan	*R*_int_ = 0.038
Absorption correction: multi-scan (*SADABS*; Sheldrick, 1996)	θ_max_ = 25.3°, θ_min_ = 3.2°
*T*_min_ = 0.928, *T*_max_ = 0.994	*h* = −7→7
20299 measured reflections	*k* = −11→11
3295 independent reflections	*l* = −19→19

### Refinement

**Table:**

Refinement on *F*^2^	Primary atom site location: structure-invariant direct methods
Least-squares matrix: full	Hydrogen site location: inferred from neighbouring sites
*R*[*F*^2^ > 2σ(*F*^2^)] = 0.053	H-atom parameters constrained
*wR*(*F*^2^) = 0.147	*w* = 1/[σ^2^(*F*_o_^2^) + (0.061*P*)^2^ + 0.4578*P*] where *P* = (*F*_o_^2^ + 2*F*_c_^2^)/3
*S* = 1.04	(Δ/σ)_max_ = 0.001
3295 reflections	Δρ_max_ = 0.29 e Å^−^^3^
221 parameters	Δρ_min_ = −0.25 e Å^−^^3^
0 restraints	

### Special details

**Table:**

Geometry. All e.s.d.'s (except the e.s.d. in the dihedral angle between two l.s. planes) are estimated using the full covariance matrix. The cell e.s.d.'s are taken into account individually in the estimation of e.s.d.'s in distances, angles and torsion angles; correlations between e.s.d.'s in cell parameters are only used when they are defined by crystal symmetry. An approximate (isotropic) treatment of cell e.s.d.'s is used for estimating e.s.d.'s involving l.s. planes.

### Fractional atomic coordinates and isotropic or equivalent isotropic displacement parameters (Å^2^)

**Table:**

	*x*	*y*	*z*	*U*_iso_*/*U*_eq_	
N2	0.2722 (3)	0.91380 (18)	0.96382 (11)	0.0383 (4)	
N1	0.8167 (3)	0.79747 (19)	0.88090 (12)	0.0420 (5)	
H1	0.8436	0.8828	0.8633	0.050*	
C8	0.6222 (4)	0.6875 (2)	0.99807 (14)	0.0452 (6)	
H8	0.7405	0.6120	1.0105	0.054*	
N3	0.0661 (3)	0.8185 (2)	1.06579 (12)	0.0480 (5)	
H3	−0.0492	0.8704	1.0424	0.058*	
O4	0.0851 (3)	0.71283 (17)	1.28015 (10)	0.0579 (5)	
C10	0.2624 (3)	0.8083 (2)	1.02536 (13)	0.0376 (5)	
C11	0.4549 (3)	0.9045 (2)	0.91869 (13)	0.0388 (5)	
H11	0.4618	0.9774	0.8752	0.047*	
O2	0.9342 (3)	0.55429 (18)	0.87934 (12)	0.0663 (5)	
N4	0.0544 (3)	0.7449 (2)	1.14426 (12)	0.0476 (5)	
H4	0.0149	0.6605	1.1495	0.057*	
C7	0.6329 (3)	0.7943 (2)	0.93254 (13)	0.0369 (5)	
O3	0.1590 (3)	0.92288 (18)	1.21135 (11)	0.0640 (5)	
C6	0.9535 (4)	0.6804 (2)	0.85656 (14)	0.0439 (6)	
C9	0.4362 (4)	0.6941 (2)	1.04441 (14)	0.0462 (6)	
H9	0.4260	0.6226	1.0884	0.055*	
C12	0.1045 (4)	0.8044 (2)	1.21198 (15)	0.0461 (6)	
C5	1.1308 (4)	0.7170 (3)	0.79916 (18)	0.0620 (7)	
H5A	1.0644	0.7759	0.7499	0.074*	
H5B	1.2121	0.7776	0.8272	0.074*	
C2	1.4473 (6)	0.7179 (4)	0.6461 (2)	0.0740 (8)	
C13	0.1179 (5)	0.7546 (3)	1.36427 (16)	0.0663 (8)	
C4	1.2840 (5)	0.5918 (4)	0.7717 (2)	0.0931 (12)	
H4A	1.2109	0.5423	0.7334	0.112*	
H4B	1.3307	0.5222	0.8199	0.112*	
O1	1.2875 (5)	0.7200 (4)	0.60720 (16)	0.1353 (12)	
C15	−0.0383 (7)	0.8914 (4)	1.3805 (2)	0.1030 (13)	
H15A	0.0014	0.9742	1.3473	0.154*	
H15B	−0.0341	0.9063	1.4388	0.154*	
H15C	−0.1825	0.8815	1.3658	0.154*	
C3	1.4820 (5)	0.6376 (5)	0.7287 (2)	0.1002 (13)	
H3A	1.5420	0.6983	0.7646	0.120*	
H3B	1.5906	0.5497	0.7237	0.120*	
C1	1.6198 (8)	0.7953 (5)	0.6113 (3)	0.1350 (18)	
H1A	1.6100	0.8071	0.5513	0.203*	
H1B	1.7593	0.7387	0.6279	0.203*	
H1C	1.6022	0.8903	0.6321	0.203*	
C16	0.0675 (7)	0.6234 (4)	1.41952 (19)	0.0943 (11)	
H16A	−0.0786	0.6116	1.4092	0.142*	
H16B	0.0821	0.6391	1.4772	0.142*	
H16C	0.1669	0.5363	1.4072	0.142*	
C14	0.3522 (7)	0.7691 (5)	1.3740 (2)	0.1111 (13)	
H14A	0.4454	0.6793	1.3617	0.167*	
H14B	0.3770	0.7881	1.4304	0.167*	
H14C	0.3832	0.8491	1.3359	0.167*	

### Atomic displacement parameters (Å^2^)

**Table:**

	*U*^11^	*U*^22^	*U*^33^	*U*^12^	*U*^13^	*U*^23^
N2	0.0429 (11)	0.0323 (10)	0.0384 (10)	−0.0052 (8)	0.0023 (8)	−0.0001 (8)
N1	0.0428 (11)	0.0303 (10)	0.0531 (12)	−0.0092 (8)	0.0086 (9)	−0.0018 (8)
C8	0.0449 (14)	0.0346 (12)	0.0516 (14)	0.0005 (10)	0.0001 (11)	0.0037 (10)
N3	0.0438 (12)	0.0504 (12)	0.0432 (11)	−0.0003 (9)	0.0058 (9)	0.0115 (9)
O4	0.0911 (13)	0.0425 (10)	0.0428 (10)	−0.0241 (9)	0.0090 (8)	0.0042 (7)
C10	0.0424 (13)	0.0334 (12)	0.0373 (12)	−0.0090 (9)	0.0000 (9)	−0.0020 (9)
C11	0.0465 (14)	0.0312 (12)	0.0383 (12)	−0.0090 (10)	0.0011 (10)	0.0016 (9)
O2	0.0826 (13)	0.0343 (10)	0.0792 (13)	−0.0052 (9)	0.0231 (10)	−0.0060 (9)
N4	0.0630 (13)	0.0354 (10)	0.0437 (11)	−0.0130 (9)	0.0095 (9)	0.0049 (9)
C7	0.0384 (12)	0.0313 (11)	0.0424 (12)	−0.0096 (9)	0.0017 (9)	−0.0046 (9)
O3	0.0948 (14)	0.0404 (10)	0.0608 (11)	−0.0279 (9)	0.0081 (10)	0.0031 (8)
C6	0.0468 (14)	0.0367 (13)	0.0488 (14)	−0.0076 (10)	0.0007 (10)	−0.0077 (10)
C9	0.0507 (14)	0.0358 (13)	0.0471 (13)	−0.0022 (10)	0.0045 (11)	0.0100 (10)
C12	0.0535 (15)	0.0349 (13)	0.0479 (14)	−0.0088 (11)	0.0110 (11)	0.0050 (11)
C5	0.0559 (16)	0.0620 (17)	0.0733 (18)	−0.0168 (13)	0.0195 (13)	−0.0244 (14)
C2	0.073 (2)	0.089 (2)	0.0620 (19)	−0.0139 (17)	0.0123 (17)	−0.0204 (17)
C13	0.096 (2)	0.0598 (18)	0.0455 (15)	−0.0248 (15)	0.0073 (14)	0.0013 (13)
C4	0.089 (2)	0.079 (2)	0.087 (2)	0.0318 (18)	0.0401 (19)	0.0168 (18)
O1	0.112 (2)	0.239 (4)	0.0606 (16)	−0.050 (2)	−0.0055 (15)	−0.0106 (19)
C15	0.154 (4)	0.075 (2)	0.080 (2)	−0.018 (2)	0.038 (2)	−0.0189 (18)
C3	0.063 (2)	0.147 (3)	0.071 (2)	0.027 (2)	0.0151 (16)	0.000 (2)
C1	0.126 (4)	0.117 (4)	0.169 (5)	−0.046 (3)	0.046 (3)	−0.009 (3)
C16	0.156 (3)	0.077 (2)	0.0518 (18)	−0.037 (2)	0.0132 (19)	0.0112 (16)
C14	0.122 (3)	0.133 (4)	0.086 (3)	−0.050 (3)	−0.025 (2)	0.011 (2)

### Geometric parameters (Å, º)

**Table:**

N2—C10	1.330 (3)	C5—C4	1.462 (4)
N2—C11	1.336 (3)	C2—O1	1.185 (4)
N1—H1	0.8600	C2—C3	1.454 (4)
N1—C7	1.409 (3)	C2—C1	1.476 (5)
N1—C6	1.339 (3)	C13—C15	1.494 (4)
C8—H8	0.9300	C13—C16	1.513 (4)
C8—C7	1.382 (3)	C13—C14	1.510 (5)
C8—C9	1.365 (3)	C4—H4A	0.9700
N3—H3	0.8600	C4—H4B	0.9700
N3—C10	1.372 (3)	C4—C3	1.517 (4)
N3—N4	1.380 (2)	C15—H15A	0.9600
O4—C12	1.336 (3)	C15—H15B	0.9600
O4—C13	1.468 (3)	C15—H15C	0.9600
C10—C9	1.386 (3)	C3—H3A	0.9700
C11—H11	0.9300	C3—H3B	0.9700
C11—C7	1.372 (3)	C1—H1A	0.9600
O2—C6	1.219 (3)	C1—H1B	0.9600
N4—H4	0.8600	C1—H1C	0.9600
N4—C12	1.333 (3)	C16—H16A	0.9600
O3—C12	1.209 (3)	C16—H16B	0.9600
C6—C5	1.495 (3)	C16—H16C	0.9600
C9—H9	0.9300	C14—H14A	0.9600
C5—H5A	0.9700	C14—H14B	0.9600
C5—H5B	0.9700	C14—H14C	0.9600
			
C10—N2—C11	117.63 (18)	O4—C13—C16	101.9 (2)
C7—N1—H1	116.9	O4—C13—C14	109.3 (3)
C6—N1—H1	116.9	C15—C13—C16	110.7 (3)
C6—N1—C7	126.27 (18)	C15—C13—C14	112.9 (3)
C7—C8—H8	120.4	C14—C13—C16	110.8 (3)
C9—C8—H8	120.4	C5—C4—H4A	109.1
C9—C8—C7	119.3 (2)	C5—C4—H4B	109.1
C10—N3—H3	120.2	C5—C4—C3	112.4 (3)
C10—N3—N4	119.59 (18)	H4A—C4—H4B	107.8
N4—N3—H3	120.2	C3—C4—H4A	109.1
C12—O4—C13	121.02 (19)	C3—C4—H4B	109.1
N2—C10—N3	114.92 (18)	C13—C15—H15A	109.5
N2—C10—C9	122.0 (2)	C13—C15—H15B	109.5
N3—C10—C9	123.07 (19)	C13—C15—H15C	109.5
N2—C11—H11	117.9	H15A—C15—H15B	109.5
N2—C11—C7	124.12 (19)	H15A—C15—H15C	109.5
C7—C11—H11	117.9	H15B—C15—H15C	109.5
N3—N4—H4	120.0	C2—C3—C4	116.5 (3)
C12—N4—N3	120.07 (19)	C2—C3—H3A	108.2
C12—N4—H4	120.0	C2—C3—H3B	108.2
C8—C7—N1	123.77 (19)	C4—C3—H3A	108.2
C11—C7—N1	118.68 (19)	C4—C3—H3B	108.2
C11—C7—C8	117.5 (2)	H3A—C3—H3B	107.3
N1—C6—C5	114.7 (2)	C2—C1—H1A	109.5
O2—C6—N1	122.5 (2)	C2—C1—H1B	109.5
O2—C6—C5	122.8 (2)	C2—C1—H1C	109.5
C8—C9—C10	119.5 (2)	H1A—C1—H1B	109.5
C8—C9—H9	120.3	H1A—C1—H1C	109.5
C10—C9—H9	120.3	H1B—C1—H1C	109.5
N4—C12—O4	109.39 (19)	C13—C16—H16A	109.5
O3—C12—O4	125.6 (2)	C13—C16—H16B	109.5
O3—C12—N4	125.0 (2)	C13—C16—H16C	109.5
C6—C5—H5A	108.3	H16A—C16—H16B	109.5
C6—C5—H5B	108.3	H16A—C16—H16C	109.5
H5A—C5—H5B	107.4	H16B—C16—H16C	109.5
C4—C5—C6	116.1 (2)	C13—C14—H14A	109.5
C4—C5—H5A	108.3	C13—C14—H14B	109.5
C4—C5—H5B	108.3	C13—C14—H14C	109.5
O1—C2—C3	121.4 (3)	H14A—C14—H14B	109.5
O1—C2—C1	120.7 (4)	H14A—C14—H14C	109.5
C3—C2—C1	117.8 (4)	H14B—C14—H14C	109.5
O4—C13—C15	110.7 (3)		
			
N2—C10—C9—C8	−1.1 (3)	C7—N1—C6—C5	178.0 (2)
N2—C11—C7—N1	−179.53 (19)	C7—C8—C9—C10	−0.6 (4)
N2—C11—C7—C8	−0.9 (3)	C6—N1—C7—C8	34.9 (3)
N1—C6—C5—C4	179.6 (3)	C6—N1—C7—C11	−146.5 (2)
N3—C10—C9—C8	176.3 (2)	C6—C5—C4—C3	−168.4 (3)
N3—N4—C12—O4	−178.94 (18)	C9—C8—C7—N1	−179.9 (2)
N3—N4—C12—O3	1.0 (4)	C9—C8—C7—C11	1.5 (3)
C10—N2—C11—C7	−0.7 (3)	C12—O4—C13—C15	59.1 (3)
C10—N3—N4—C12	82.6 (3)	C12—O4—C13—C16	176.9 (2)
C11—N2—C10—N3	−175.89 (18)	C12—O4—C13—C14	−65.8 (3)
C11—N2—C10—C9	1.7 (3)	C5—C4—C3—C2	−70.8 (4)
O2—C6—C5—C4	−0.5 (4)	C13—O4—C12—N4	−176.3 (2)
N4—N3—C10—N2	−160.78 (19)	C13—O4—C12—O3	3.8 (4)
N4—N3—C10—C9	21.6 (3)	O1—C2—C3—C4	−15.7 (5)
C7—N1—C6—O2	−1.8 (4)	C1—C2—C3—C4	165.3 (3)

### Hydrogen-bond geometry (Å, º)

**Table:**

*D*—H···*A*	*D*—H	H···*A*	*D*···*A*	*D*—H···*A*
N1—H1···O3^i^	0.86	2.06	2.888 (2)	161
N3—H3···N2^ii^	0.86	2.21	2.957 (3)	145
N4—H4···O2^iii^	0.86	2.06	2.827 (3)	149

Symmetry codes: (i) −*x*+1, −*y*+2, −*z*+2; (ii) −*x*, −*y*+2, −*z*+2; (iii) −*x*+1, −*y*+1, −*z*+2.
